# Patient lived experiences of functioning and disability following lumbar discectomy: a secondary analysis of qualitative data

**DOI:** 10.1186/s12891-024-07790-7

**Published:** 2024-09-12

**Authors:** Jai Mistry, Louise White, Karl Baraks, Chris Davis, Pulak Parikh, Siobhan Schabrun, Nicola Heneghan, Tim Noblet, David Walton, Alison Rushton

**Affiliations:** 1 School of Physical Therapy, Faculty of Health Sciences, Western University, London, ON Canada; 2 St Georges Hospital NHS Foundation Trust, London, UK; 3 Queen Elizabeth Hospital Birmingham, Physiotherapy, Birmingham, UK; 4 Physiotherapy Department, Sandwell and West Birmingham Hospitals Trust, Birmingham, UK; 5 Centre of Precision Rehabilitation for Spinal Pain, University of Birmingham, Birmingham, UK

**Keywords:** Patient, Experience, Functioning, Disability, Lumbar discectomy

## Abstract

**Background:**

Knowledge of patient lived experiences of functioning and disability is limited. This study aims to address the gap in the literature by exploring patient lived experiences of functioning and disability following lumbar discectomy.

**Method:**

A secondary analysis, reported in line with the Standards for Reporting Qualitative Research, was conducted of qualitative data exploring patient journeys following lumbar discectomy surgery (DiscJourn). Adult patients (≥ 16 years) undergoing elective or emergency primary lumbar discectomy were recruited from one National Health Service secondary care centre in the UK. Semi-structured interviews were conducted at 1–3 weeks and 1-year post surgery. Participants who completed both semi-structured interviews were eligible for the secondary analysis. Transcripts from the semi-structured interviews were analysed using interpretative phenomenological analysis (IPA). IPA involved two independent reviewers identifying themes for individual data sets followed by an iterative process involving the wider research team to identify overarching themes that represented the whole date set. Subthemes generated from the IPA were mapped against the International Classification of Functioning, Disability and Health (ICF) framework at the level of chapters, in order to ascertain the ICF’s utility in capturing experiences of functioning and disability. Strategies to enhance trustworthiness of data analysis included blind coding, peer examination and debrief, declaration of pre-conceived beliefs and active reflexivity throughout the study.

**Results:**

Nine participants met the eligibility criteria and their interview transcripts were analysed. Patient lived experiences of functioning and disability were captured by three overarching themes: Immediate impact following surgery, Multiple roads to recovery over 1 year, and Functioning influenced by personal loci of control. Each theme consisted of three subthemes which were subsequently mapped onto the ICF. Three subthemes mapped to the ICF’s body component, 1 to activity and participation and 3 to environment. Two subthemes themes did not map onto the ICF.

**Conclusion:**

Findings provide valuable insights into patient experiences of functioning and disability following lumbar discectomy. Convergence in experiences of functioning and disability were identified immediately following surgery. Divergence in such experiences were identified with regards to the roads to recovery over 1 year and the individuals’ locus of control. Findings build on the body of literature exploring patients functioning and disability following discectomy and make recommendations for future research and clinical practice.

## Background

Low back-related leg pain (LBLP) has gained increasing interest in contemporary research due to the great burden it places on the individual and to society [1]. Neuropathic pain (NP) is commonly reported in those with LBLP with recent prevalence estimates ranging between 48 and 74% [2]. A common presentation of NP in LBLP is when the disc has been implicated in pain emanating from the nerve root by means of mechanical distortion or biochemical irritation [3]. Surgery is indicated for those describing disabling intractable NP LBLP and/or worsening neurology (loss of power and/or sensation) in the distribution of the implicated nerve root/s [4]. The most common surgery in cases of LBLP where the disc has been identified as the primary cause of pain emanating from the nerve root is a discectomy [5].

In the western world spinal surgeries have become increasingly common [6], with discectomies the most common spinal surgery in both the USA [7] and UK [8]. In the UK alone, 1688 primary lumbar discectomies were performed annually in 2021–2022 in the National Health Service (NHS) [8], with average cost per surgery estimated to be at £6200 [9]. In order to justify the significant financial burden discectomies put on healthcare systems, post-surgery outcomes are of great interest. Discectomy success rates, when observed across multiple studies, range from 46 to 75% at 6–8 weeks [10–12], and 78–95% at 1–2 years post-surgery [10–15]. The clinical course of pain and disability following primary lumbar discectomy have recently been described in a systematic review and meta-analysis. Findings from the 87 included studies identified clinically relevant improvements in pain (measured by the visual analogue scale (VAS)) and disability (measured by the Oswestry Disability Index (ODI) immediately post discectomy and at long term follow up (7 years) [16].

Despite the reports of high success rates and a favourable clinical course following lumbar discectomy, ongoing post-surgical issues across bio-psycho-social domains of health have been reported, including persistent pain [17], motor deficits [18], work related dissatisfaction [19] and lower quality of life scores [18] for some patients. The patient reported outcome measures (PROMs) used to determine success rates and clinical course following discectomy largely seek to capture information regarding the patient’s function (SF-36 – physical functioning/social functioning) and disability (ODI, Roland Morris Disability Questionnaire (RMDQ)) status. However, PROMs used to capture broad, and arguably unmeasurable constructs such as function and disability have been found to lack content validity as the experiences explored may not capture the construct in its entirety [20]. For this reason, ‘success’ rates following discectomy may not accurately reflect patients’ experiences of functioning and disability.

The ICF framework is recognised as an international standard for the conceptualisation of functioning and disability [21]. The ICF framework adopts a biopsychosocial model and conceptualises functioning and disability as a dynamic interaction between a person’s health condition, environmental factors and personal factors [22]. However, it has been argued that at its heart the ICF still subscribes to a biomedical conceptualisation of disability and fails to capture the individual experience of functioning and disability [23]. To date no research has investigated patient experiences of functioning and disability following lumbar discectomy using the ICF as a framework to characterise these experiences, therefore its utility is unknown. To enhance our understanding of surgical ‘success’ rates and variable outcomes amongst discectomy patients, function and disability must be explored beyond patient reported outcome measures [24]. Only patients themselves can describe their unique experiences. Therefore, it is imperative that research investigating patient experiences of functioning and disability post discectomy is explored.

This study seeks to address the gap in the literature by exploring patient lived experiences of functioning and disability following lumbar discectomy through qualitative analysis of interview transcripts. Additionally, the study will critically evaluate the ICF’s utility in capturing patients experiences of functioning and disability.

### Aim

To explore patient experiences of functioning and disability over 1 year following lumbar discectomy.

### Objectives

#### Primary

1. To analyse interview transcripts using IPA and to generate themes that represent patient post-operative experiences of functioning and disability over 1 year.

#### Secondary

2. To map subthemes generated from IPA against the ICF framework and critically evaluate its utility in capturing experiences of functioning and disability.

## Methods

### Theoretical framework

An interpretative phenomenology framework was adopted using IPA to explore patient experiences of functioning and disability following lumbar discectomy. Interpretative phenomenology subscribes to the Heideggerian notion that lived experience can be understood through interpretive processes [25]. The researcher cannot be separated from the researched. This framework is underpinned by an interpretivist theoretical perspective and thus aligns with the positionality of the lead author of this study.

### Study design

A secondary analysis of qualitative data using an IPA approach and reported in line with the Standards for Reporting Qualitative Research [26]. Data were collected from a primary study titled ‘DiscJourn’ [24]. White et al. [24] aimed to explore patients’ experiences of their lumbar discectomy journey through weekly diaries and semi structured interviews (at 1–3 weeks and 1 year post op).

**Fig. 1:**
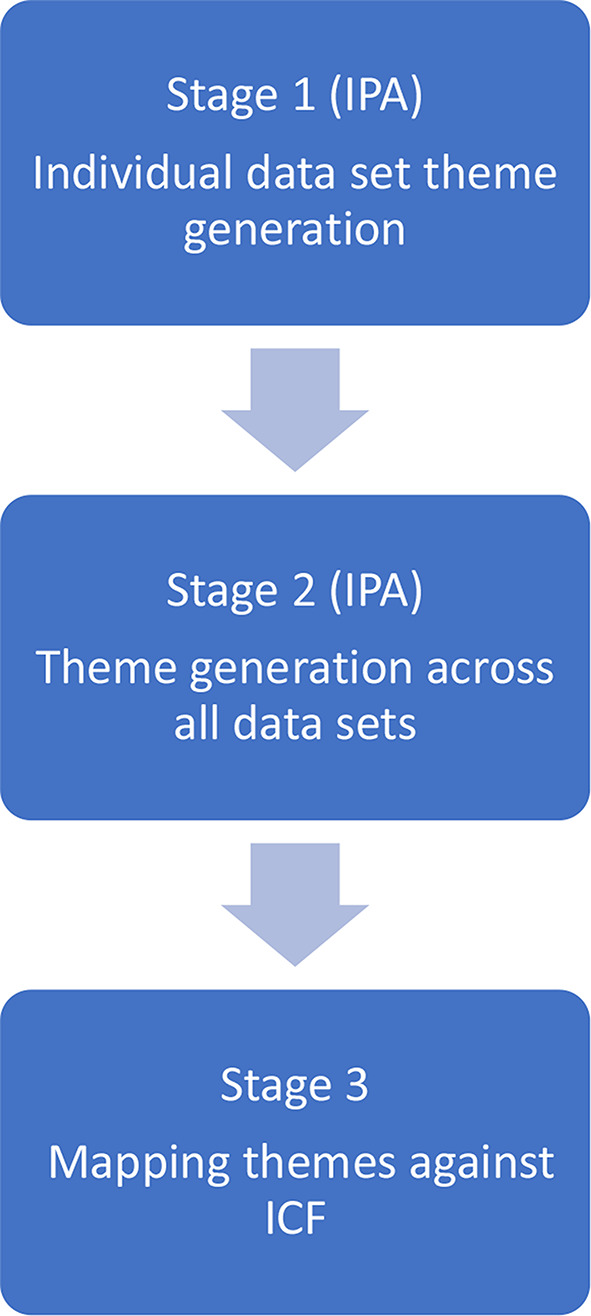
Study design

This secondary analysis used IPA to explore patient experiences of functioning and disability. IPA was developed by Smith [27] in the field of psychology and has since become one of the dominant qualitative research methodologies in health care research [28]. It provides structure through a framework whilst also maintaining a flexible inductive approach [29]. IPA is phenomenological in that it describes lived experience however it also subscribes to hermeneutics as it recognises interpretation as essential when understanding lived experiences [27]. Case by case analysis is essential in IPA where an idiographic approach is encouraged in contrast to a nomothetic approach. Only once each case has been examined, does the researcher look for convergence or divergence between cases [27]. The study design is described in Fig. 1.

The study design consisted of 3 stages. IPA was conducted within stages 1 and 2 and mapping onto the ICF was conducted in stage 3.

Stage 1: Themes were generated for each data set using IPA. To date there is no previous research exploring patient experiences of functioning and disability following lumbar discectomy, therefore an inductive interpretive approach enabled themes to be derived directly from the transcripts.

Stage 2: The themes generated from each individual data set in stage 1 were brought together to interpret superordinate themes to represent the whole data set. Themes were observed for convergence and divergence, the goal being to identify ‘higher level’ themes at this stage to represent theoretical convergence whilst maintaining the idiographic nuance identified in stage 1 [27].

Stage 3: The subthemes generated from stage 2 were mapped against the ICF framework. The utility of the ICF to capture the subthemes generated from the IPA was considered with a critical lens.

### Ethics

Ethical approval for this secondary analysis was obtained through Western University, Canada (Review Reference: 2022-121383-73782). Permission to access the data was supported by the DiscJourn study sponsor, the University of Birmingham, UK. A data sharing agreement between the University of Birmingham and Western University was in place. Confidentiality was maintained throughout this study by allocating an ID code to each participant and not including any identifiable information.

### Participants

In the primary DiscJourn study a purposive sample of 14 participants was recruited from a single secondary care setting in the UK (Queen Elizabeth Hospital, University Hospitals Birmingham NHS Foundation Trust). Participants were identified by staff members from a neurosurgery team [24]. In the secondary analysis a purposive subsample was selected from the participants who met the eligibility criteria for the DiscJourn study. Eligibility criteria for DiscJourn and this secondary analysis are depicted in Table 1.

**Table 1:** Eligibility criteria for DiscJourn and secondary analysis

**DiscJourn**	
Inclusion	Adults (≥ 16 years old) undergoing elective or emergency primary lumbar discectomy surgery.
Exclusion	Malignancy, infection, poor English or communication difficulties.
**Secondary Analysis** (selected from eligible DiscJourn participants)	
Inclusion	DiscJourn Participants who completed both semi structured interviews (1–3 weeks and 1-year post surgery) for which transcripts are available.

### Data collection

The primary DiscJourn study collected data using weekly (written or electronic) diaries and transcripts from semi-structured interviews conducted at 1–3 weeks and 1 year post operatively [24]. In the secondary analysis, data were analysed from the interviews alone, as they provide a greater depth of data compared to the diaries [30].

### Interviews

Interviews were conducted twice, by the principal investigator (PI) (LW) of DiscJourn or second investigator (KB), over the first post-operative year; at 1–3 weeks and 12 months post-surgery. Interviews were either conducted at the hospital site or participant homes based on participant preference. Interview transcripts were informed by previous systematic reviews, parallel study investigating lumbar fusion and the authors of the DiscJourn study [24]. Using a topic guide [24], the interviewer encouraged participants to explore issues pertinent to them to capture their ‘individual journey.’ Following the first interview the authors (LW, NH, NF, AM, KB, AR) of DiscJourn adapted the topic guide to inform the follow up interview at 12 months based on the analysis of a participant’s first interview. A reflective diary and field notes were recorded by the interviewer. Interviews were audio recorded and professionally transcribed verbatim. Participants were given the opportunity to read and approve their transcripts and add further reflections if required.

For the secondary analysis both interviews were analysed to capture the longitudinal nature of the post-operative experiences of functioning and disability. Longitudinal qualitative data provide insights into how and why experiences change over time. The complexity of experiences can be explored in greater detail at multiple time points and therefore a more realistic understanding of the lived experience can be achieved [31]. Additionally, a familiarity with the interview process can lead to participants feeling more comfortable during the second interview leading to richer experiences being described [32].

### Data storage

In the original DiscJourn study data were stored in accordance to the Data Protection Act (DPA) and the General Data Protection Regulation (GDPR) (2018). For the secondary analysis a data sharing agreement was agreed with the University of Birmingham to share the data with Western University. Following ethical approval from Western University (WREM ref: 2022-121383-73782) data were stored on OneDrive and access was granted to the Chief Investigator of the original study (AR) and the primary researcher of the secondary analysis (JM).

### Data analysis

#### Data were analysed in three distinct stages

##### Stage 1

The ICF’s broad definition of functioning and disability was adopted; ‘Functioning and disability are understood as umbrella terms denoting the positive and negative aspects of functioning from a biological, individual and social perspective’ [33]. The definition operationalised the terms functioning and disability which is essential for data analysis, whilst still remaining broad in nature which allowed for researcher interpretation. Two investigators (JM and CD) read and re-read transcripts to familiarise themselves with the data. Both investigators initially analysed the transcripts independently. Investigators analysed both the 1–3 week and 1-year transcripts together for each participant to form a data set. Investigators used the left-hand margin of each transcript to note points of interest or significance related to participants experiences of functioning and disability. The right-hand margin was used to describe emerging title themes. The developed title themes were brought together and clusters of similar themes were developed. Finally, the clusters of themes were captured in overarching superordinate themes that represented the themes for each data set. The two investigators convened after the generation of superordinate themes for each data set to discuss and compare findings in an iterative process [34].

##### Stage 2

The same two investigators independently collated the superordinate themes for each individual data set agreed upon in stage 1 and looked for superordinate themes of similar concepts to represent the whole data set [35]. The process involved re-examining the superordinate themes for each individual data set, going back to the transcripts, peer examination and debrief. Once both investigators agreed upon superordinate themes across the whole data set, these themes were presented to the wider research team. A similar process of discussion, challenging assumptions and beliefs and returning to the original transcripts was conducted with the wider research team. Following consensus agreement, the resultant superordinate themes represented researcher interpretations of participants’ experiences of functioning and disability following lumbar discectomy [36].

##### Stage 3

Subthemes generated from the IPA (stages 1 and 2) were mapped against the ICF framework at the level of chapters. Mapping of subthemes at the level of chapters was agreed upon by the research team. ICF chapters are both broad and descriptive which was deemed appropriate for subtheme mapping [37]. Subthemes that did not fit within the ICF were defined as emergent subthemes. This stage was conducted by the lead investigator (JM) and brought to the wider research team to discuss findings. The ICF was critically evaluated by observing how many subthemes mapped onto the framework and by exploring how well the ICF captured the meaning of the subthemes.

### Trustworthiness

To enhance trustworthiness, themes were generated independently by the two investigators and then compared [38]. Peer examination and debrief, by the wider research team, was used to critically explore interpretations involved in theme generation [38]. Investigators declared their pre-conceived assumptions and beliefs and considered the impact this may have on data analysis, additional actively reflexivity was present throughout the study through discussion with peers and self-reflection. The adoption of a ‘reflexive and curious’ attitude was set as a precedent for data analysis to enhance receptiveness to alternative perspectives [36].

Both research investigators were PhD students of health sciences at the University of Western, Ontario. Clinically, both investigators work as Advanced Practice Physiotherapist in the UK, one in public health the another in the private sector. Both investigators have experience in the treatment of patients following discectomy and both have experience in qualitative research methods. Both investigators were not involved in the original DiscJourn study.

### Patient and public involvement

Patient involvement was central throughout the whole research process for the DiscJourn study. Two patients known to the DiscJourn team contributed to development of the interview topic guide, patient diaries proforma, participant information sheet, consent form, and data analysis of the parent study [24].

## Results

Nine participants met the eligibility criteria for the study. See participant characteristics in Table 2.

**Table 2:** Characteristics of participants

Characteristic	Category of characteristic	*n* (%)
Age (years)	35–44	5 (55.5%)
	45–54	1 (11.1%)
	55–64	1 (11.1%)
	65–74	1 (11.1%)
	> 75	1 (11.1%)
Sex	Female	4 (44.4%)
	Male	5 (55.5%)
Ethnicity	White British	8 (88.8%)
	British Asian	1 (11.1%)
Surgical procedure	Discectomy	2 (22.2%)
	Microdiscectomy	6 (66.6%)
	Discectomy and decompression	1 (11.1%)
Level of surgery	L4/5	4 (44.4%)
	L5/S1	5 (55.5%)
Elective or emergency	Elective	7 (77.7%)
	Emergency	2 (22.2%)
Symptom duration	0–1 year	3 (33.3%)
	1–2 years	4 (44.4%)
	> 2 years	2 (22.2%)
Employment status	Employed/self employed	6 (66.6%)
	Unemployed	1 (11.1%)
	Retired	2 (22.2%)
Co-existing past medical history	Depression	2 (22.2%)
	Cardiac	1 (11.1%)
	Nil	6 (66.6%)

Three superordinate themes were identified, representing participants’ experiences of functioning and disability following lumbar discectomy over 1 year. These included (1) Immediate impact following surgery, (2) Multiple roads to recovery over 1 year, and (3) Functioning influenced by personal sense of locus of control. These themes are described further below including illustrative verbatim quotes.

### Theme 1: Immediate impact following surgery

A unanimous experience described by all participants was an immediate, positive, impact following their surgery. The immediate impact largely pertained to improvement in pre-operative symptoms. Three sub-themes characterised theme 1:

- *Immediate improvement in leg pain and neurological symptoms.*
- *Immediate improvement in physical ability.*
- *Impact of surgery exceeding expectation.*

Subtheme descriptions and illustrative quotations depicted in Table 3.

**Table 3:** Subthemes for immediate impact following surgery

Subtheme	Description	Illustrative quotations
*Immediate improvement in leg pain and neurological symptoms*	All participants described a marked improvement in leg pain and neurological symptoms immediately after surgery. Improvements in leg pain seemed to be the more impactful change amongst participants. Although neurological symptoms persisted for some, they were greatly improved compared to pre-operation.	*“I’m great now. I’ve got no pain. I still have*,* like I said*,* my leg still goes numb now and again but that’s generally if I’ve sat there too long” (P7)* *“As I got out the bed and that*,* immediately I realised that the pain had completely gone” (P10)*
*Immediate improvement in physical ability*	Most participants described an immediate improvement in physical ability, largely pertaining to mobility. Correlation with improvements in pain and neurological symptom was a key component to the improvement in physical ability.	*“So I was actually walking. I was up and about soon*,* right after surgery” (P2)* *“As I got out of bed and went to walk*,* I went to drag my leg and the pain wasn’t there. I was like… I’ll just try walking normally and it was fantastic.” (P2)*
*Impact of surgery exceeded expectations*	Participants commonly reported their expectations were exceeded immediately after surgery. Again, this was largely due to the significant reduction in leg pain as well as reduction in neurological symptoms.	*“Interviewer: Having been for your surgery*,* do you feel it’s met your expectations?’* *Respondent: More than.” (P13)* *“On my part it far exceeded my expectation” (P4)*

### Theme 2: multiple roads to recovery over 1 year

Roads to recovery were multifactorial and complex and varied amongst participants over 1-year post surgery. Individual roads to recovery were informed heavily by how a participant perceived their spine following surgery. Two conceptual perceptions were highlighted, which extended across all subthemes: normal/new acceptable normal and vulnerable. ‘Normal’ spines were characterised by those who at 1-year post-operation described their spinal health as no longer a limiting factor in their physical function. ‘New acceptable normal’ spines were characterised by participants who believed that post-operatively their spine has changed and is somewhat limited compared to baseline. However, the limitations are of little functional consequence and thus ‘acceptable’. ‘Normal’ and ‘New acceptable normal’ perceptions have been grouped together as experiences of functioning and disability were closely aligned. Finally, ‘vulnerable’ spines were perceived by those who believed their spine to be fragile or at risk of damage leading them to avoid activities and limit their function to protect from further injury.

Three sub-themes characterised theme 2:

- *Ongoing back and neurological symptoms had varying effects on functioning over 1 year.*
- *Fear of re-injury/pain driven by biomedical understanding of symptoms.*
- *Recovery dependent on access to health care professionals (HCP) input.*

Subtheme descriptions and illustrative quotations depicted in Table 4.

**Table 4:** Subthemes for multiple roads to recovery over 1 year

Subtheme	Description	Illustrative quotations
*Ongoing back and neurological symptoms had varying effects on functioning over 1 year*	Ongoing back pain and neurological symptoms over the post-operative year was common amongst participants, however the extent of pain/symptoms and the effect on functioning varied. Participants who identified with a ‘vulnerable’ spine generally described limitations to recovery due to pain and ongoing neurological symptoms.	*“Yeah*,* I do a lot less now which is not great. I spend a lot of time inside. I would love to be doing a lot more things*,* but I don’t last very long. You know I go out and I manage like an hour. And then I’m having to call time out and say guys*,* I can’t do anything anymore. I’m too sore.” (P5)*
	Those who identified with a ‘normal’/‘new acceptable spine’ described upward trajectories of recovery throughout the post-operative year despite ongoing pain and neurological symptoms.	*“I still get backache*,* but it is what it is” (P4)* *“So*,* slowly*,* I’m getting a lot better and I’m doing a lot more things. And my pain’s not come back. I still have the odd aches and pains now and again but I can cope with that.” (P7)*
*Fear of re-injury driven by biomedical understanding of symptoms*	Fear of re-injury was described by many participants at some point in their post-operative recovery. However, the extent to which the fear acted as a barrier to recovery varied greatly. Participants commonly described fear of re-injury, pertaining to wound health, particularly during the early stages of post-operative recovery. Those who identified with a ‘normal’/’new acceptable normal’ spine, if fearful, tended to increase in confidence following the early post-operative phase and were not fearful throughout the remaining post-operative year.	*“Right*,* okay. One of the scariest things for me*,* I’ve found*,* was getting in and out of bed… because now I’ve had my staple things out*,* I’m scared of opening the wound up.” (P2)* *“I do a little bit of washing up and making cups of tea and things like that*,* very light duties but as I’ve said to you*,* if I’ve got to reach for anything and I feel any tenderness or pulling or anything which is not quite right*,* I don’t*,* I stop immediately” (P13) (1st interview at 1–3 weeks)* *“You’re not cautious now at all?* *Respondent: Oh no*,* no.” (P13) (2nd interview at 1 year)*
	Fear persisted in those who identified with a ‘vulnerable’ spine. Fear of structural re-injury significantly affected functioning throughout post-op year.	*“I think that the more active I am*,* the more worse it gets because they’re grinding together so then it’s making them disappear altogether” (P7)* *“So I’m not putting weight – I don’t want to put pressure on one side of my body – on my spine – I’m trying to even the weight out. So just protecting myself really. I don’t want to have surgery again*,* or any issues with my back” (P9)*
*Recovery dependent on access to HCP input*	Access to Health Care Professionals (HCP) throughout post year varied amongst participants. With a common complaint being a lack of timely access to see a HCP post operatively. Generally, it was deemed the more input from HCPs throughout the post-operative year the better.	*“…I think it’s that aftercare where*,* like I just said*,* you need to see the surgeon a bit earlier*,* you need physio a bit earlier…” (P6)* *“I had to go on a waiting list to do the physio*,* which I think I shouldn’t have had to do. It should have been available straight away” (P9)* *“I think*,* in terms of help*,* you know*,* it’s helpful to*,* you know*,* meet with [HCP name] and talk things through at different times” (P1)*
	Those who identified with a ‘vulnerable’ spine tended to require more input from HCPs. A lack of input was a barrier to functioning. A reason for needing to see HCPs links to the fear of re-injury and requiring reassurance from HCPs.	*“I would still like somebody to talk to*,* who could still be give out the advice. Even if it’s a phone call away*,* somebody” (P8)* *“I sort of limit myself to what I do. Because I haven’t been back to see him” (P7)*

### Theme 3: functioning influenced by individual’s locus of control

A participant’s locus of control (internal and external) greatly influenced functioning. Three subthemes characterised theme 3:

- *Optimism and ability to ‘get on with it’ helped functioning.*
- *Social support both facilitator and barrier to functioning.*
- *Reliance on HCPs for permission to engage in activity.*

Subtheme descriptions and illustrative quotations depicted in Table 5.

**Table 5:** Subthemes for functioning influenced by individual’s locus of control

Subtheme	Description	Illustrative quotations
*Optimism and ability to ‘get on with it’ helped functioning*	Optimism and ability to ‘get on with’ were traits described by participants who generally recovered well over the post-operative year. The two were not mutually exclusive however both were deemed as favourable traits. Optimism was commonly associated with the immediate improvements following surgery.	*“Interviewer: You’re feeling pretty optimistic and positive. Respondent: Definitely*,* 100%.” (P13)* *“I think generally I’m all positive at this point because the achievement that we’ve had*,* having this procedure*,* gives me a light at the end of the tunnel*,* which I haven’t had. This is like seriously moving forward for me.” (P5)* *“I’d made my mind up I was going to recover come hell or high water. I’m that kind of mentality.” (P4)* *“Yeah*,* and worked at my own pace and was back at work after two weeks. So yeah*,* had to just get on with it” (P5)*
*Social support both facilitator and barrier to functioning*	Social support was described by participants in a variety of ways. Dichotomously social support was largely perceived as a barrier or facilitator. Those who received social support from family, friends, carers etc… generally found this to help recovery and facilitate functioning. However, in some instances support can be a barrier due to an over-protective support network.	*“It was the first day*,* yesterday they (Carers) came*,* I’ve got them for*,* I think it’s five to six weeks*,* while I’m in my recovery period. I think that’s a must. That’s definitely a must for anyone that has this type of operation*,* they need some form of care” (P2)* *“…because motivation*,* I’ve got none at the minute. Inside*,* I’m fine. You know*,* I do general housework and things like that. But they [family] sort of have to come and get me to say*,* “Oh come on*,* we’re going so-and-so.” Because if it’s left to me*,* then I’m quite happy to stop in the house all day” (P7)* *“Interviewer: Am I right in saying that you now feel that you could do a wee bit more than you are at this stage but your wife is putting the reins on you at the moment.* *Respondent: Yeah.” (P13)*
	A lack of social support was not reported by any participant. However, some participants reported a preference to going through their recovery alone, despite access to social support.	*“Interviewer: Do you think that your family and friends helped to keep you going? Do you feel that you were quite self-sufficient? Respondent: Self*,* definitely self-sufficient*,* yes.” (P13)* *“And now – even now – I’m by myself. I’m looking at my health. No-one’s going to look after my health as in say to me*,* “Go to the gym*,*” and check what I’m doing.” I’m there. I’ll go to the gym by myself. I train by myself. I’m there for myself. So it’s more about me” (P9)*
*Reliance on HCPs for permission to engage in activity*	Reliance on HCPs to give permission to engage in activity was commonly reported. This was noted particularly during the early post-operative stage.	*“Then I think that I’ll start to move on with my life then. because it sort of permission I think to say*,* “Yeah*,* everything is great. Continue with life as normal.” But at the minute*,* I sort of limit myself to what I do. Because I haven’t been back to see him because I don’t know how it’s worked or how it’s not worked.” (P7)*

### Mapping onto the ICF

**Fig. 2:**
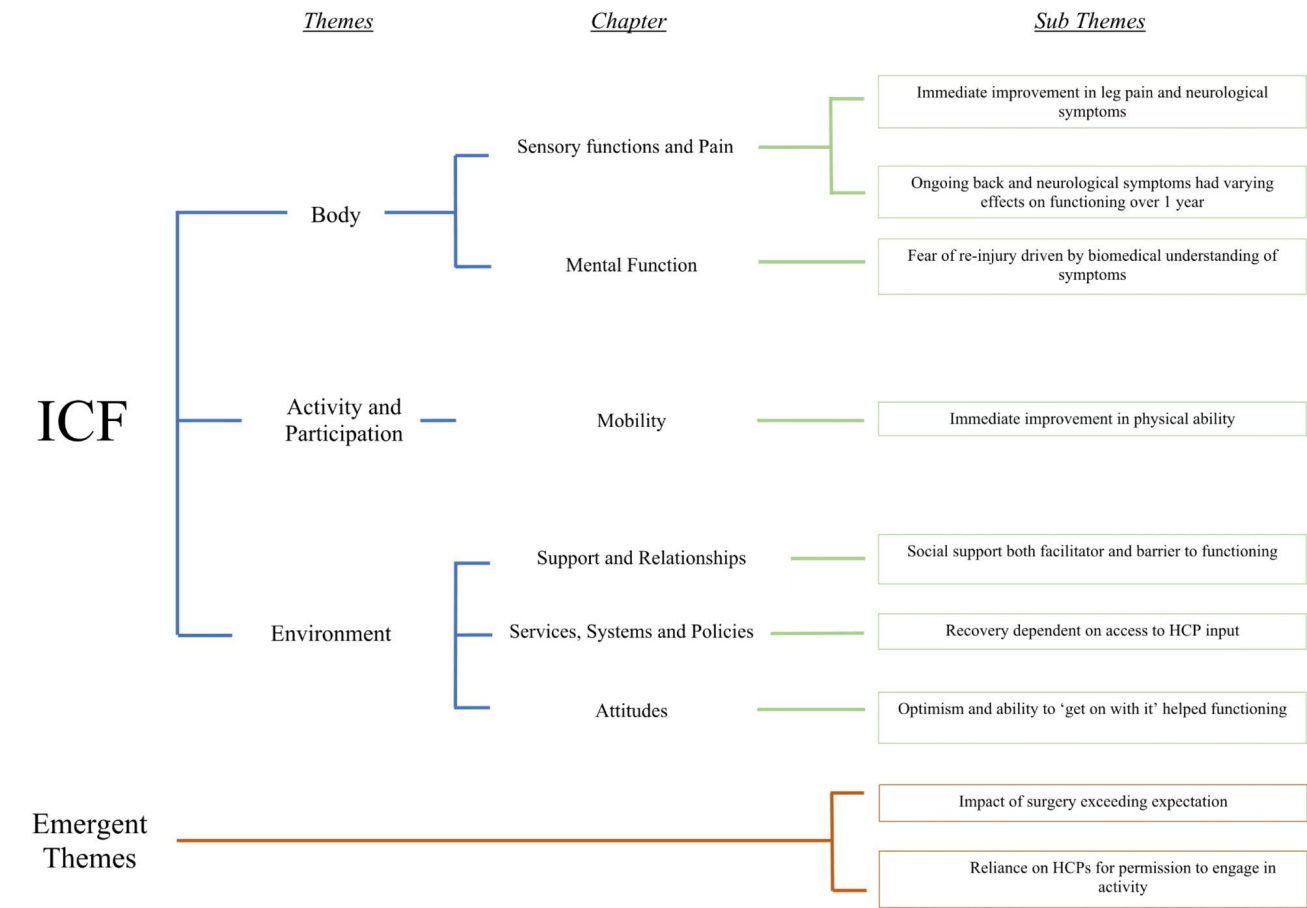
ICF mapping

Of the 9 subthemes identified, 7 mapped onto the ICF and 2 were described as emergent as they did not directly map. Three subthemes were mapped within the ICF’s Body Structure and Function construct, 1 within the Activity and Participation construct and 3 within the Environment construct. Mapping is depicted in Fig. 2. The 7 subthemes mapped onto the ICF did not retain their meaning when mapped e.g., the subtheme ‘Immediate improvement in leg pain and neurological symptoms’ when mapped onto the ICF was described as “Sensory functions and pain”.

### Body function and structure

Three sub-themes mapped onto the ICF at the level of body, specifically body function. Two subthemes mapped to the chapter ‘Sensory functions and Pain’: *Immediate improvement in leg pain and neurological symptoms* and *Ongoing back and neurological symptoms had varying effects on functioning over 1 year*. One subtheme mapped to the ICF chapter ‘Mental Function’: *Fear of re-injury driven by biomedical understanding of symptoms.*

### Activity and participation

One subtheme mapped onto the ICF at the level of Activity and Participation. The subtheme mapped to the ICF chapter ‘Mobility’: *Immediate improvement in physical ability.*

### Environment

Three subthemes mapped onto the ICF at the level of Environment. One subtheme mapped to the ICF chapter ‘Support and Relationships’: *Social support both facilitator and barrier to functioning.* Another subtheme mapped to the ICF chapter ‘Services, Systems and Policies’: *Recovery dependent on access to HCP input.* The final subtheme mapped to the ICF chapter ‘Attitudes’: *Optimism and ability to ‘get on with it’ helped functioning.*

### Emergent themes

Two subthemes were not mapped onto the ICF. These themes were described as emergent themes: *Impact of surgery exceeding expectations* and *Reliance on HCPs for permission to engage in activity.*

## Discussion

This is the first study to explore patient experiences of functioning and disability following lumbar discectomy. Three broad overarching themes were identified representing patients’ experiences: ‘Immediate impact following surgery’, ‘Multiple roads to recovery over 1 year’ and ‘Functioning influenced by individual’s locus of control’. These findings align with previous research with regards to the ‘Immediate impact following surgery’ [10, 16] however the varying experiences of functioning and disability highlighted in ‘Multiple roads to recovery over 1 year’ and ‘Functioning influenced by individual’s locus of control’ are novel. These findings when interpreted alongside PROMs defining clinical course and ‘success’ following discectomy add a greater depth of understanding to patients’ functioning and disability experiences.

### Immediate impact following surgery

A unanimous experience of functioning amongst all participants in this study, was that of an immediate positive response to surgery. Particularly, participants described the reduction in leg pain to be of great significance improving physical ability. These findings are supported by results from a systematic review investigating the clinical course of pain and disability following lumbar discectomy, where immediate clinically relevant improvements in leg pain and disability were identified [16]. The immediacy of the changes following discectomy surgery is of great interest, and mirror findings from individuals undergoing lumbar fusion surgery [39]. Participants commonly described the impact of surgery to exceed their expectations, which builds on research investigating patient expectations following spinal surgery for degenerative spinal conditions, where 80% of patients report their expectations were met following surgery [40]. Expectations can influence a multitude of different factors, however the authors of this study hypothesised that the exceeding expectations of the impact of surgery described by participants in the secondary analysis can be attributed, in part, to; the selection of appropriate patients for surgery, the expectations set by the surgeon and the significant improvement in debilitating leg symptoms. Understanding of the experiences of functioning immediately after surgery present an opportunity for patients and HCPs to capitalise on at the early post-operative stage.

### Multiple roads to recovery over 1 year

Functioning and disability experiences over 1 year were individualised and multifactorial amongst participants. The research team identified two conceptual perceptions held by participants of their spines’ following surgery which largely shaped their functioning and disability experiences over 1 year; ‘normal’/’‘new acceptable normal’ and ‘vulnerable.’ Ongoing pain and numbness throughout the post-operative year was reported, however, the extent to which this affected functioning and disability was variable. Machado et al. [17] found patients with sciatica experience mild to moderate pain levels up to 5 years post-surgery, however it is unclear from their findings what effect pain had on recovery. In the current study, participants whose functioning was limited due to ongoing pain and neurological symptoms tended to identify with a ‘vulnerable’ spine. Whereas others, despite ongoing pain and neurological symptoms continued to function, in keeping with a good recovery trajectory, these participants were those who identified with a ‘normal’/’new acceptable normal’ spine. Fear of re-injury was commonly described amongst participants particularly at the early post-operative stage. Fear was associated with the structural integrity of the spine becoming compromised, with participants using language concerning their discs such as; ‘slip’, ‘bulge’ and ‘wear’. The understanding of their spine and the language associated with it seemed to manifest at the pre-operative stage in which diagnostic tests and HCP’s explanations of symptoms and surgical procedures occurred. It could be argued that an overly bio-medicalised understanding of symptoms may lead to fear evoking behaviour [41]. However, it could equally be argued that based on the high rates of re-incidence of disc herniation and subsequent re-operation that a medical understanding of symptoms is useful to limit harmful behaviours, in that some fear at the early post-operative stages may be useful [42]. Fear of re-injury did not persist for all participants, however for those where it did, function was hampered significantly. The participants who remained fearful throughout the post-op year were those of a ‘vulnerable’ spine disposition. Evidence suggests that fear avoidant behaviours can lead to the failure of spinal surgery, and pre-operative identification of fear can help to identify patients who would benefit from psychological intervention post operatively [43]. A lack of access to HCPs post-operatively was generally deemed a barrier to function by most participants. Despite the information provided in leaflets and the advice given prior to discharge, participants described the need to see an HCP sooner to ask questions concerning what they can and cannot do and questions regarding their symptoms. These findings draw similarity to those experienced by patients following lumbar spinal fusion who describe a feeling of ‘abandonment’ post operatively due to the lack of contact with HCPs [39]. Despite the ‘Getting it Right First Time’ (GIRFT) recommendations for review with a physiotherapist at 2–3 weeks following discectomy, most participants had to wait much longer [44]. Most participants were happy to be reviewed by any HCP involved in their care however some, those who were particularly fearful and worried of structural re-injury to their spine, were keen to be reviewed by the neurosurgeon. Ongoing pain and neurological symptoms, fear of re-injury driven by a biomedical understanding of symptoms and lack of access to HCPs were generally deemed as barriers to functioning, particularly in those who identified with a ‘vulnerable’ spine.

### Functioning influenced by individual’s locus of control

Patient experiences of functioning and disability were heavily influenced by their locus of control (internal and external). Optimism and the ability to ‘get on with it’ were identified as traits that had a positive influence on functioning post-surgery. Optimism is a known predictor of physical health and has been demonstrated to have a negative association with pain [45]. Those who described optimistic feelings carried this with them throughout their post-operative journey. Some participants described optimism immediately after their operation, this could be partly explained by the immediate positive impact following surgery. Others described optimism at the pre-operative stage when they were under the neurosurgeon and knew they were due to have surgery; perceiving light at the end of the tunnel. A good patient-clinician relationship has been found to have significant correlation with feelings of optimism and hope, highlighting the importance of pre-operative and post-operative interactions with HCPs [46]. Social support was generally reported as favourable amongst participants, particularly in relation to engaging with physical activity. Perceived social support and recovery are positively associated [47]. However, in some instances social support was not required and even avoided as participants preferred to go through the recovery journey independently. Also, some participants found being overly ‘supported’ was a barrier to functioning. Evidence surrounding social support following surgery is conflicting with evidence for and against its utility, the findings from the secondary analysis align with this conflict as social support varied dependent on the needs of the individual [48]. Reliance on HCPs for permission to engage in activity was described by some participants, particularly those who were fearful and in need of reassurance. The long wait to see an HCPs resulted in some participants not engaging with physical activity until late into their post-operative journey. A general dissatisfaction with generic advice and exercise was described by some participants, who tended to favour individualised care, could explain the need to want to see someone in person. A preference towards individualised care has been demonstrated in patients with non-specific low back pain (NSLBP) in the RESTORE trial and linked to better health related outcomes [49]. However, this was not consistent amongst all participants and likely the need for permission was related to a need for reassurance. Understanding of a patient’s unique locus of control can help clinicians to identify aspect of care which can be focused on which in turn can facilitate functioning.

### ICF utility in capturing patients experiences of functioning and disability

Patient experiences of functioning and disability were found to relate to the body, activity and participation and environment components of the ICF. The descriptive and interpretive nuances in the subthemes were only partially captured by the broad descriptive ICF chapters. Although many of the descriptive terms (e.g. fear) were represented by the ICF, the meaning behind the themes were lost when mapped. Furthermore, two themes, related to the concepts of permission and expectation, were not featured when mapped to the ICF. If the ICF framework was used in this study as a conceptual framework to characterise experiences of functioning and disability it would do so at the cost of the rich underlying meaning behind the experiences. Although the ICF is considered an international standard for describing and understanding functioning and disability, in the case of capturing experiences of functioning and disability in this study, it was insufficient. Despite the iteration made from its predecessor, the ICF at its core still subscribes to a positivist theoretical perspective where conceptualisation of functioning and disability is funnelled into pre-defined categories and codes [23]. The nature of seeking to understand experiences aligns itself to an interpretivist perspective and therefore a disparity between the themes generated in this study and the ICF framework becomes clear at a philosophical level.

## Strengths and limitations

A key strength of this study was as a secondary analysis it reduced the burden on participants for research participation as the transcripts were already available [50]. Furthermore, the use of IPA allowed themes to be generated through a process of describing as well as seeking to understand the underlying meanings of participants experiences. However, a limitation of IPA is as it is idiographic it lacks external validity. Another strength to this study was multiple strategies were employed to enhance the trustworthiness of this study during the data analysis stage. A limitation was the participants were recruited from a single UK site and thus may not be representative of the wider population. The primary author of the secondary analysis was not involved in the primary DiscJourn study and therefore was not involved in data collection and thus unable to be fully immersed in the data. However, this could be seen as a strength as without influencing the data the author approached it without preconceived notions and beliefs of the data and its meaning [50].

### Recommendations for further research and clinical implications

In this study we used only transcripts from semi-structured interviews to identify patient experiences of functioning and disability however the primary DiscJourn study also collected data in the form of weekly diary entries. Research investigating patient experiences of functioning and disability using the diary entries may provide new insights into this area. Furthermore, research investigating pre surgery and long term follow up (> 1 year) experiences of functioning and disability will build from the findings of this study and complete the discectomy journey. Study findings are not generalisable to the entire discectomy population due to the idiographic nature of IPA. However, the findings provide clinicians with valuable insights into patients experience of functioning and disability following discectomy. Particularly the divergence in experiences signify the importance of treating the individual based on their unique needs.

## Conclusion

Patient experiences of functioning and disability over 1 year following lumbar discectomy were captured in 3 overarching themes. Convergence in experiences of functioning and disability were identified with regards to the immediate impact following surgery. Divergence in experiences of functioning and disability were identified with regards to the roads to recovery over 1 year and the individuals’ loci of control. Divergence in experiences highlighted the multifaceted, complex and unique nature of patient experiences of functioning and disability. These findings can be interpreted alongside the existing body of literature exploring functioning and disability following discectomy to add to our current understanding of success rates and clinical course.

## Data Availability

No datasets were generated or analysed during the current study.
